# Internal doses in experimental mice and rats following exposure to neutron-activated ^56^MnO_2_ powder: results of an international, multicenter study

**DOI:** 10.1007/s00411-020-00870-x

**Published:** 2020-09-29

**Authors:** Valeriy Stepanenko, Andrey Kaprin, Sergey Ivanov, Peter Shegay, Kassym Zhumadilov, Aleksey Petukhov, Timofey Kolyzhenkov, Viktoria Bogacheva, Elena Zharova, Elena Iaskova, Nailya Chaizhunusova, Dariya Shabdarbayeva, Gaukhar Amantayeva, Arailym Baurzhan, Bakhyt Ruslanova, Zhaslan Abishev, Madina Apbassova, Ynkar Kairkhanova, Darkhan Uzbekov, Zaituna Khismetova, Yersin Zhunussov, Nariaki Fujimoto, Hitoshi Sato, Kazuko Shichijo, Masahiro Nakashima, Aya Sakaguchi, Shin Toyoda, Noriyuki Kawano, Megu Ohtaki, Keiko Otani, Satoru Endo, Masayoshi Yamamoto, Masaharu Hoshi

**Affiliations:** 1 Medical Radiological Research Center named after A.F. Tsyb-branch of “National Medical Research Center of Radiology” Ministry of Health of the Russian Federation, Koroleva Str. 4, Obninsk, 249036 Kaluga Russian Federation; 2 National Medical Research Center of Radiology, Ministry of Health of the Russian Federation, Koroleva Str. 4, Obninsk, 249036 Kaluga Russian Federation; 3 Eurasian National University named after L.N. Gumilyov, Astana, 2 Satpayev Str., Nur-Sultan, 010000 Republic of Kazakhstan; 4 Semey Medical University, 103 Abay Str., Semey, 071400 Republic of Kazakhstan; 5 Research Institute for Radiation Biology and Medicine, Hiroshima University, 1-2-3, Kasumi, Minami-ku, Hiroshima, 734-8551 Japan; 6 Ibaraki Prefectural University of Health Sciences, 4669-2 Ami-chyo Ami, Inashiki-gun, Ibaraki, 300-0394 Japan; 7 Atomic Bomb Disease, Institute, Nagasaki University, 1-12-4, Sakamoto, Nagasaki 852-8102 Japan; 8 Faculty of Pure and Applied Sciences, University of Tsukuba, 1-1-1, Tsukuba-shi Tennodai, Ibaraki, 305-8571 Japan; 9 Department of Applied Physics, Okayama University of Science, 1-1 Ridai, Kita-ku, Okayama, 700-0005 Japan; 10 The Center for Peace, Hiroshima University, Higashisenda-machi 1-1-89, Naka-ku, Hiroshima, 730-0053 Japan; 11 Graduate School of Engineering, Hiroshima University, 1-4-1, Kagamiyama, Higashi, Hiroshima 739-8527 Japan; 12 Graduate School of Natural Science and Technology, Kanazawa University, Kakuma-Cho, Kanazawa, 920-1192 Japan

**Keywords:** ^56^Mn, Neutron activation, Dispersion of radioactivity, Radioactive dust, Internal irradiation, Experimental mice and rats

## Abstract

The experiment was performed in support of a Japanese initiative to investigate the biological effects of irradiation from residual neutron-activated radioactivity that resulted from the A-bombing. Radionuclide ^56^Mn (T_1/2_ = 2.58 h) is one of the main neutron-activated emitters during the first hours after neutron activation of soil dust particles. In our previous studies (2016–2017) related to irradiation of male Wistar rats after dispersion of ^56^MnO_2_ powder, the internal doses in rats were found to be very inhomogeneous: distribution of doses among different organs ranged from 1.3 Gy in small intestine to less than 0.0015 Gy in some of the other organs. Internal doses in the lungs ranged from 0.03 to 0.1 Gy. The essential pathological changes were found in lung tissue of rats despite a low level of irradiation. In the present study, the dosimetry investigations were extended: internal doses in experimental mice and rats were estimated for various activity levels of dispersed neutron-activated ^56^MnO_2_ powder. The following findings were noted: (a) internal radiation doses in mice were several times higher in comparison with rats under similar conditions of exposure to ^56^MnO_2_ powder. (b) When 2.74 × 10^8^ Bq of ^56^MnO_2_ powder was dispersed over mice, doses of internal irradiation ranged from 0.81 to 4.5 Gy in the gastrointestinal tract (small intestine, stomach, large intestine), from 0.096 to 0.14 Gy in lungs, and doses in skin and eyes ranged from 0.29 to 0.42 Gy and from 0.12 to 0.16 Gy, respectively. Internal radiation doses in other organs of mice were much lower. (c) Internal radiation doses were significantly lower in organs of rats with the same activity of exposure to ^56^MnO_2_ powder (2.74 × 10^8^ Bq): 0.09, 0.17, 0.29, and 0.025 Gy in stomach, small intestine, large intestine, and lungs, respectively. (d) Doses of internal irradiation in organs of rats and mice were two to four times higher when they were exposed to 8.0 × 10^8^ Bq of ^56^MnO_2_ (in comparison with exposure to 2.74 × 10^8^ Bq of ^56^MnO_2_). (e) Internal radiation doses in organs of mice were 7–14 times lower with the lowest ^56^MnO_2_ amount (8.0 × 10^7^ Bq) in comparison with the highest amount, 8.0 × 10^8^ Bq, of dispersed ^56^MnO_2_ powder. The data obtained will be used for interpretation of biological effects in experimental mice and rats that result from dispersion of various levels of neutron-activated ^56^MnO_2_ powder, which is the subject of separate studies.

## Introduction

Our experiments were performed in support of a Japanese initiative to investigate the biological effects of irradiation from residual neutron-activated radioactivity that resulted from the A-bombing (Hoshi 2020). During nuclear explosions that take place in the atmosphere, neutron-activated radionuclides are distributed in surface layers of the soil, contributing to the beta and gamma irradiation that results from residual radioactivity. The main radionuclides are ^24^Na, ^28^Al, ^31^Si, ^32^P, ^38^Cl, ^42^K, ^45^Ca, ^46^Sc, ^56^Mn, ^59^Fe, ^60^Co, and ^134^Cs (Weitz 2014). Radionuclide ^56^Mn (T_1/2_ = 2.58 h) is one of the main neutron-activated emitters during the first hours after neutron activation of soil dust particles (Tanaka et al. 2008; Weitz 2014). The purpose of this international multicenter study was to extend our previous work (Shichijo et al. 2017; Stepanenko et al. 2017) to estimate internal doses for laboratory animals (mice and rats) with different exposures to ^56^MnO_2_ in the form of dispersed powder. The results of the internal dose assessments will be used to investigate the biological effects that result from this type of exposure, which will be the subject of future publications.

## Materials and methods

Table 1 gives details of the laboratory mice and rats used in the experiments and also the initial ^56^Mn activity (100 mg ^56^MnO_2_ powder sprayed over the animals while they were in their cages).

**Table 1:** Laboratory mice and rats (supplier: Kazakh Scientific Center of Quarantine and Zoonotic Diseases, Almaty, Kazakhstan under contract with Charles River Laboratories, Germany) and initial activity of neutron-activated ^56^MnO_2_ powder used for spraying over animals in each cage)

Date of exposure	Laboratory animals	Initial^56^Mn activity in 100 mg ^56^MnO_2_ powder used for spraying over the animals in each cage**)
17.08.2018	CD-1 mice, 11-week-old male	2.74 × 10^8^ Bq
17.08.2018	Wistar rats, 11-week-old male	2.74 × 10^8^ Bq
18.08.2018	Wistar rats, 11-week-old male	5.5 × 10^8^ Bq
18.08.2018	Wistar rats, 11-week-old male	8.0 × 10^8^ Bq
22.04.2019	C57BL mice, 10-week-old male	8.0 × 10^8^ Bq
22.04.2019	C57BL mice, 10-week-old male	2.74 × 10^8^ Bq
23.04.2019	C57BL mice, 10-week-old male	8.0 × 10^7^ Bq
17.06.2019	C57BL mice, 10-week-old male	2.74 × 10^8^ Bq
17.06.2019	BALB/C mice, 10-week-old male	2.74 × 10^8^ Bq
18.06.2019	C57BL mice, 10-week-old male	8.0 × 10^8^ Bq
18.06.2019	BALB/C mice, 10-week-old male	8.0 × 10^8^ Bq

As a result of irradiation of 100 mg MnO_2_ by thermal neutrons with fluence *F* = 1.2 × 10^14^ neutron/cm^2^, the yield of ^56^Mn activity (Ao) is equal to 8 × 10^7^ Bq. The ratio Ao/*F* is equal to 6.7 × 10^−7^ Bq per thermal neutron/cm^2^

**Numbers of animals in each cage for each irradiation were different—from 6 to 9 (rats) and from 3 to 10 (mice) animals per cage

The total numbers of mice and rats targeted for dosimetry only were 24 and 9, respectively. Along with the animals scheduled for dosimetry, animals that were intended for subsequent biological studies were additionally placed in the same cages. As a result, the total number of animals in each cage for each irradiation was different, from 6 to 9 rats and from 3 to 10 mice per cage.

**Fig. 1:**
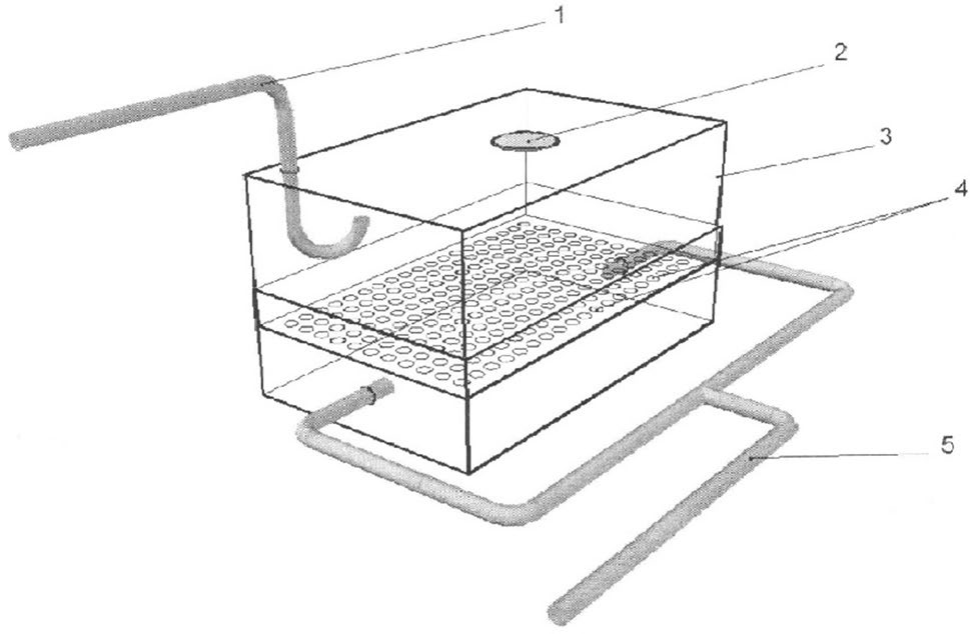
Schematic view of the box where neutron-activated radioactive ^56^MnO_2_ powder was dispersed on experimental animals. 1-Pneumatic tube for dispersion of radioactive ^52^Mn powder; 2-air filter; 3-plastic wall of the box; 4-plastic floor of the box with holes, where experimental animals were placed; 5-tubes for forced ventilation

**Fig. 2:**
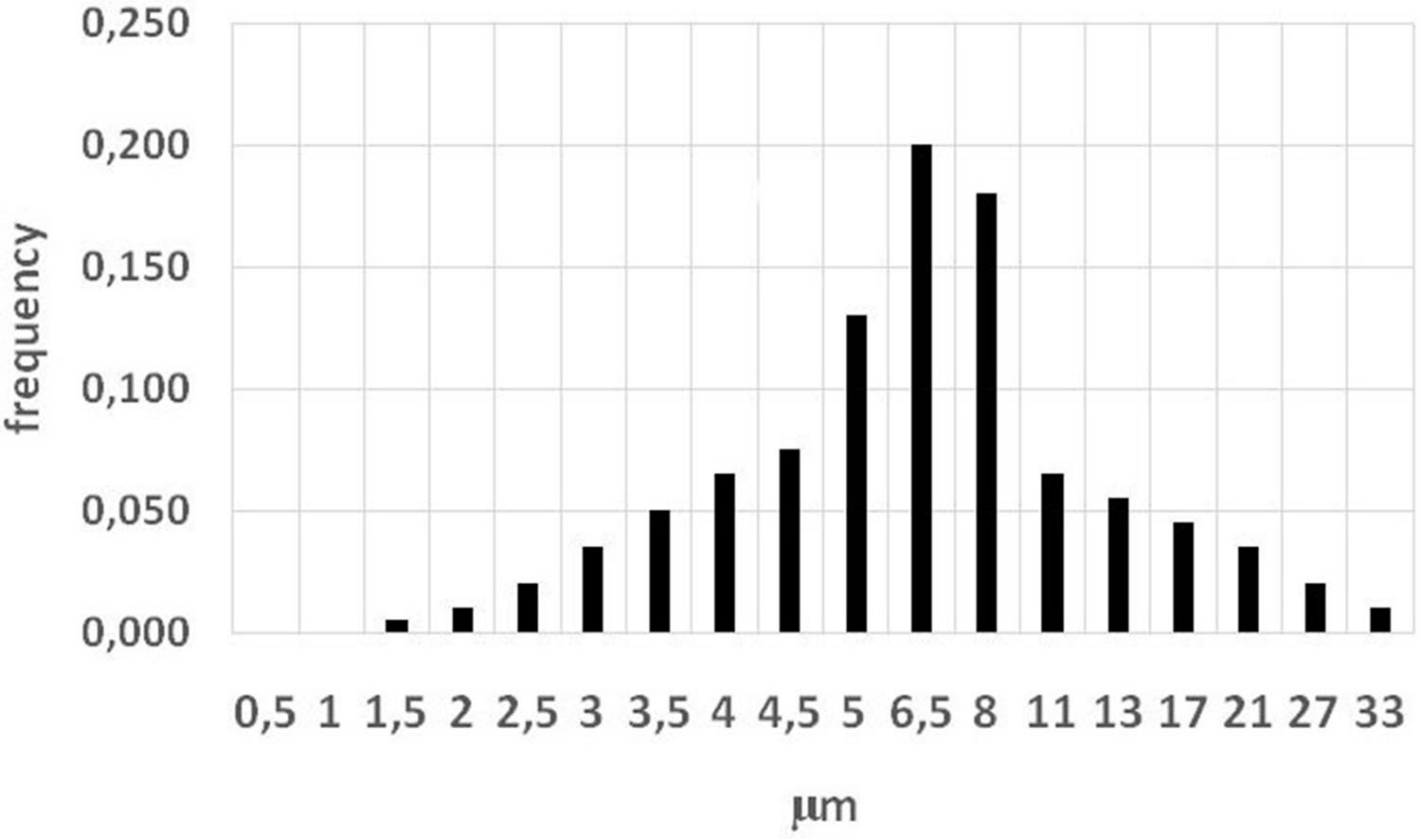
Statistical distribution of MnO_2_ particle diameters. Vertical axis: frequency, relative units. Horizontal axis- diameter of particles, µm. Mean diameter of MnO_2_ particles is equal to 8.1 µm

All experimental work was performed during 2018–2019 at research reactor IVG.1 (“Baikal-1”) located in the territory of the Semipatinsk nuclear test site (Lanin 2013), Republic of Kazakhstan. Details of neutron activation of MnO_2_ powder (Rare Metallic Co., Ltd) and exposure of animals to dispersed ^56^MnO_2_ particles were presented in our previous paper (Stepanenko et al. 2017). Briefly, experimental animals were placed in special boxes for exposure to ^56^MnO_2_ powder (Fig. 1). One hundred milligrams of activated powder was used for each ^56^MnO_2_ exposure. Statistical distribution of MnO_2_ particle sizes is presented in Fig. 2. Animals were exposed for 1 h. Exposed animals were removed from cages and euthanized by injection of an excessive dose of pentobarbital. All work with experimental animals was approved by the ethics committee of Semey State Medical University, Kazakhstan, according to directive 2010/63/EU of the European Parliament and the Council of the Office on protection of animals used for scientific purposes of 22 September 2010 (Directive 2010/63/EU 2010). The following organs and tissues were surgically extracted from experimental animals: lungs, heart, small intestine, large intestine, stomach, esophagus, liver, spleen, kidney, trachea, skin, eyes, and blood. To measure specific activity of ^56^Mn, small pieces (about 1 ml) of each organ were weighed and subjected to gamma-spectrometry by an AMPTEC, Inc., Gamma-Rad5 spectrometer with an NaI(Tl) detector. Details of measurement conditions and calibration of the spectrometer were presented in our previous paper (Stepanenko et al. 2017). A description of internal dose estimations according to the Medical Internal Radiation Dose methodology (Bolch et al. 2009) was presented in the same paper. According to MIRD methodology, internal radiation doses were assessed by taking into account accumulated activity of ^56^Mn in all studied organs (which are listed above), self-irradiation of these organs, and their irradiation by all other sampled organs and tissues. Calculation of absorbed fractions of energy in studied organs from beta and gamma irradiation of ^56^Mn was performed using the Monte-Carlo method (Briemeister 2000) and age-dependent mathematical phantoms of rats and mice (Stepanenko et al. 2015). The spectrum of ^56^Mn beta particles (Stabin et al. 2001) was accounted for internal dose calculations. Gamma irradiation from ^56^Mn (Be et al. 2013) was accounted for as well.

## Results

**Fig. 3:**
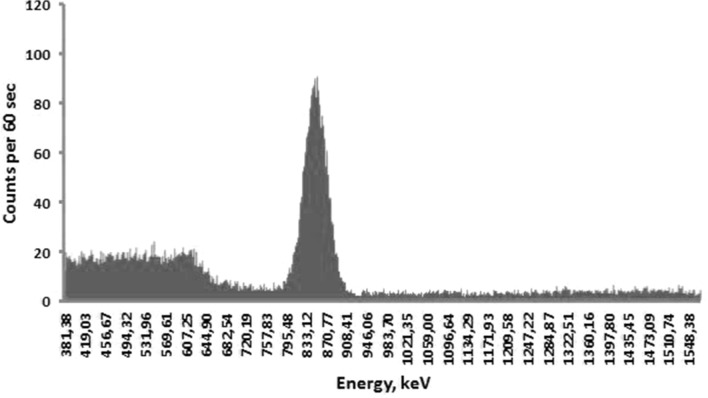
Gamma spectrum of ^56^Mn obtained from the sample of the lung of a mouse. The maximal peak corresponds to the 846.8 keV gamma energy (intensity—98.9%) of ^56^Mn. Background gamma spectrum measured in the shielded room was subtracted

**Fig. 4:**
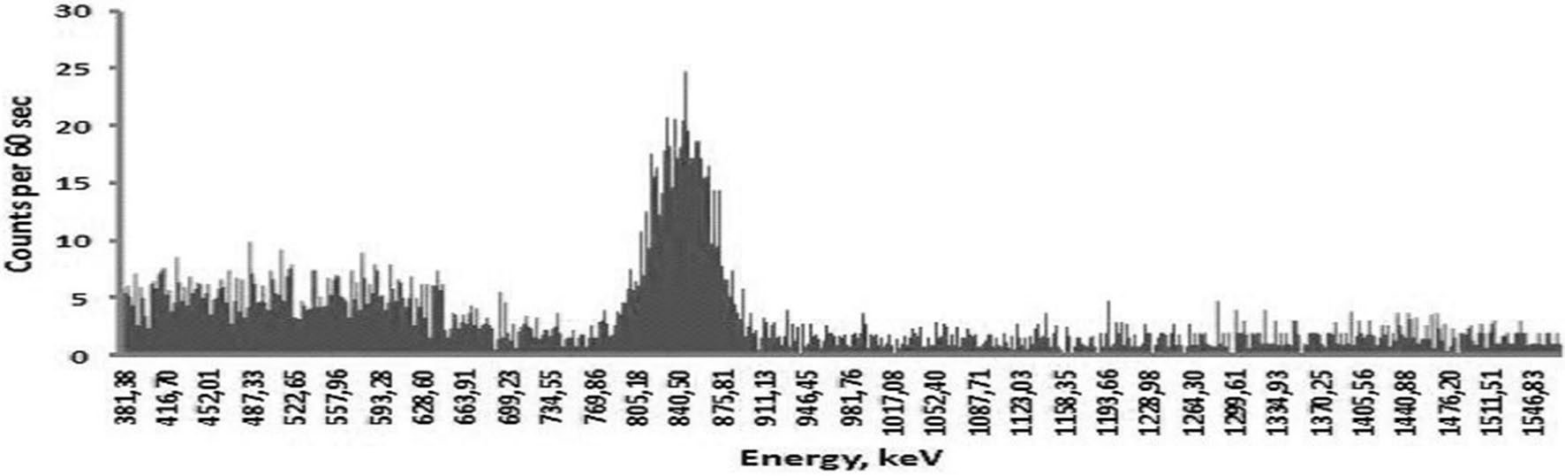
Gamma spectrum of ^56^Mn obtained from the sample of the lung of a rat. The maximal peak corresponds to the 846.8 keV gamma energy (intensity—98.9%) of ^56^Mn. Background gamma spectrum measured in the shielded room was subtracted

**Fig. 5:**
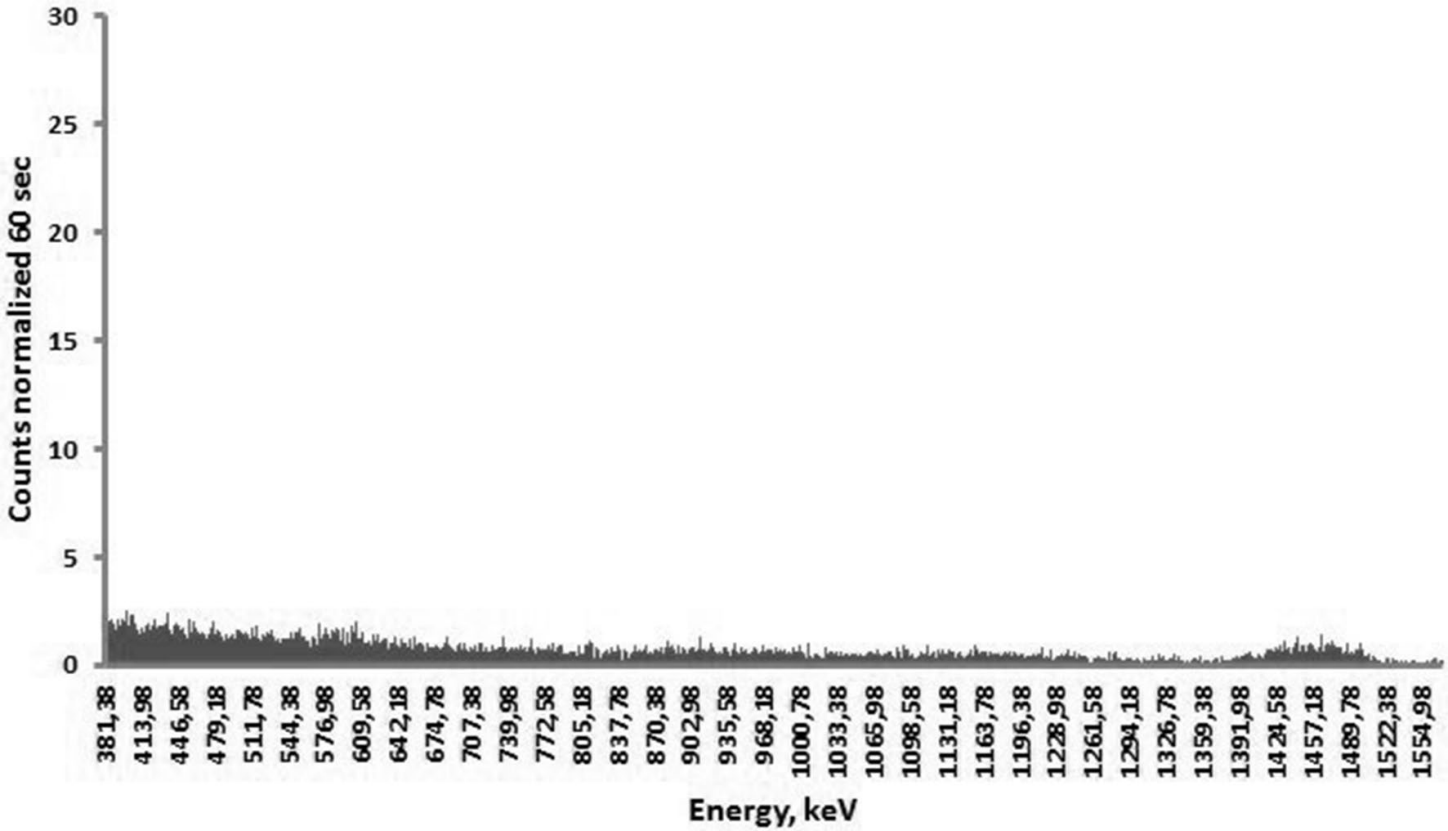
Background gamma spectrum measured in a well-shielded “measuring lab” without any radioactive samples

Each extracted sample of organs (lungs, heart, small intestine, large intestine, stomach, esophagus, liver, spleen, kidney, trachea, skin, eyes, and blood) from all investigated laboratory animals was subjected to gamma spectrometry in a well-shielded room. Volumes of extracted samples were small enough (about 1 ml) to consider them as radiating point sources in comparison with distance to and size of the spectrometer’s detector. The highest ^56^Mn specific activities were found in large and small intestine, stomach, lungs, and skin, which corresponds to our previous results obtained from similar experiments on rats (Stepanenko et al. 2017). A typical gamma spectra of ^56^Mn measured by a gamma-spectrometer are presented in Figs. 3 and 4. In both examples with measured gamma spectrum of ^56^Mn in biological samples, the amount of ^56^MnO_2_ powder dispersed over the experimental animals was equal. The background gamma spectrum measured in a well-shielded room inside the reactor building is presented in Fig. 5.

**Fig. 6:**
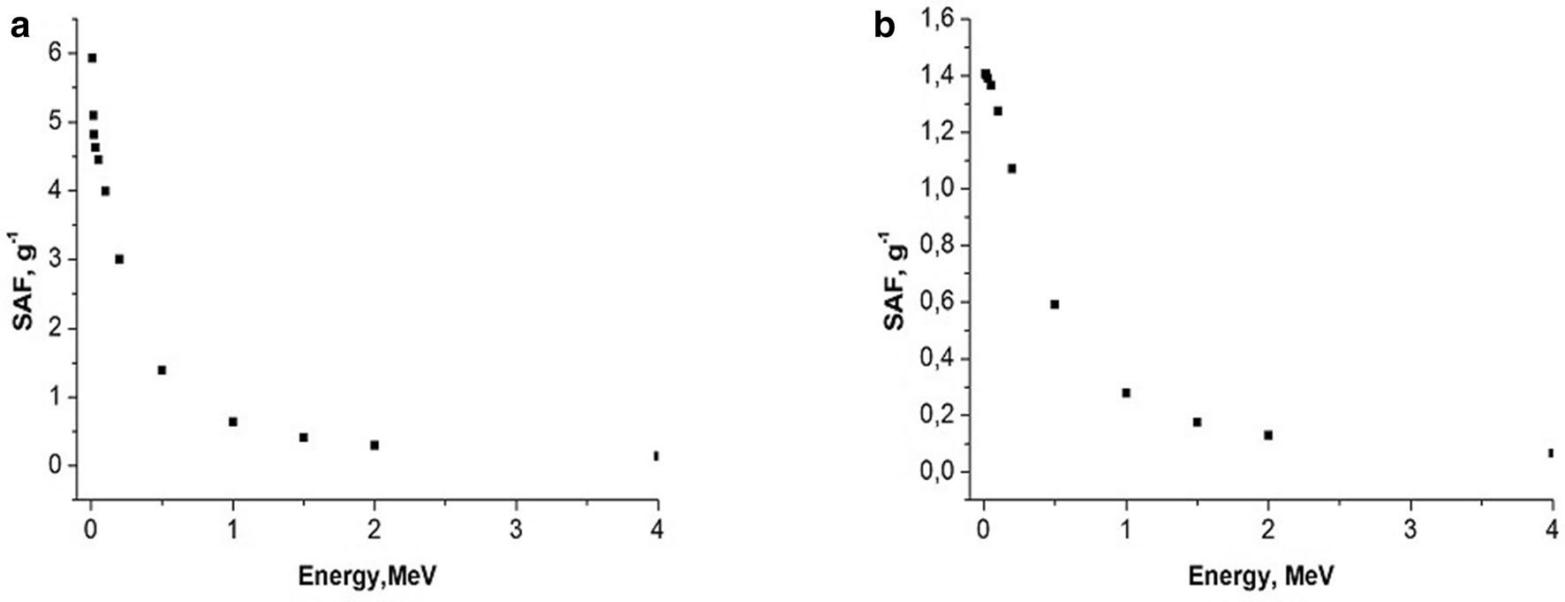
Self-irradiation of lungs by electrons as a function of energy, MeV. Left panel: mouse, right panel: rat. Whole body weight of mouse (**a**): 30 g; whole body weight of rat (**b**): 270 g. SAF, g^−1^: specific absorbed fraction of electron energy

**Fig. 7:**
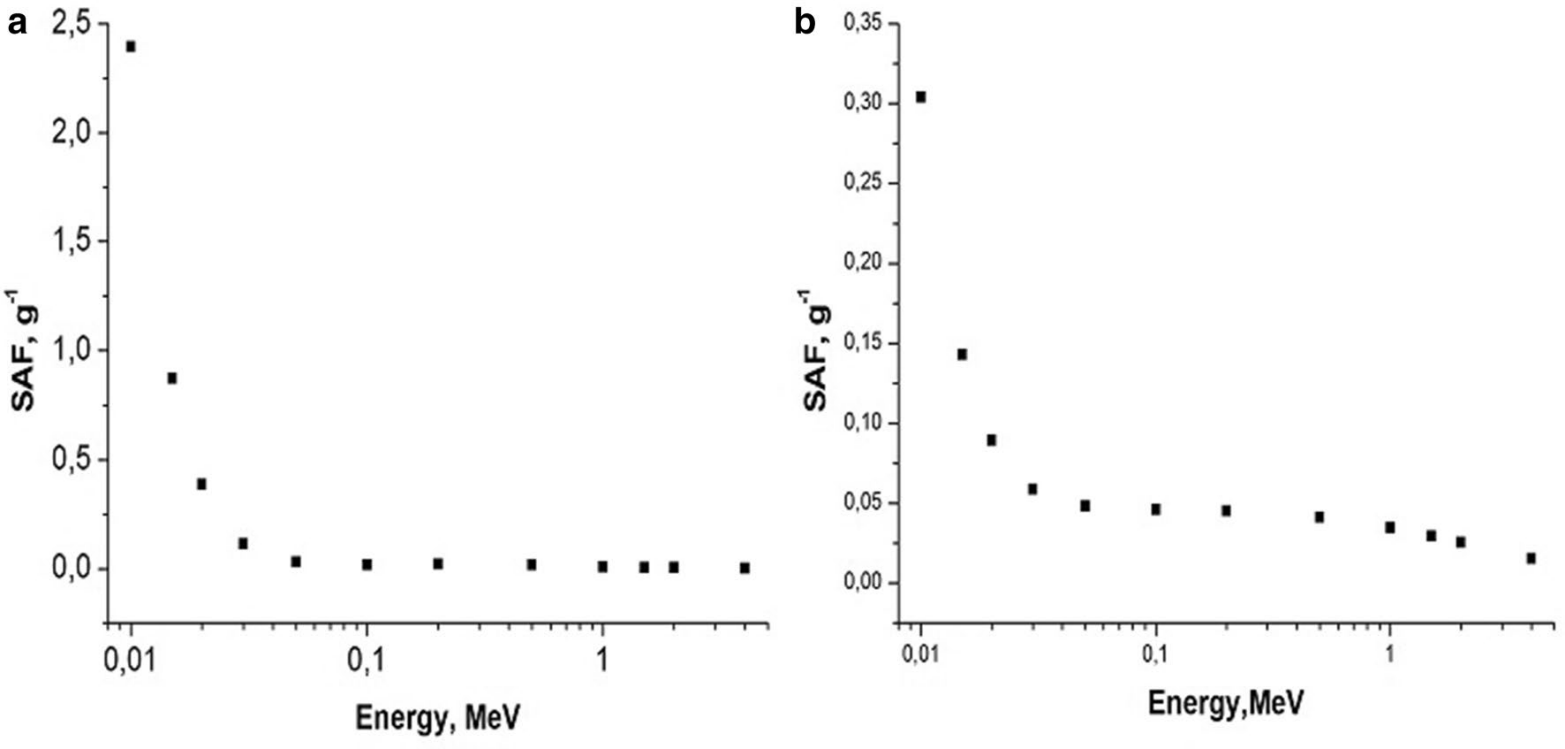
Self-irradiation of lungs by gammas as a function of energy, MeV. Left panel: mouse, right panel: rat. Whole body weight of mouse (**a**): 30 g; whole body weight of rat (**b**): 270 g; SAF, g^−1^: specific absorbed fraction of gamma energy

**Fig. 8:**
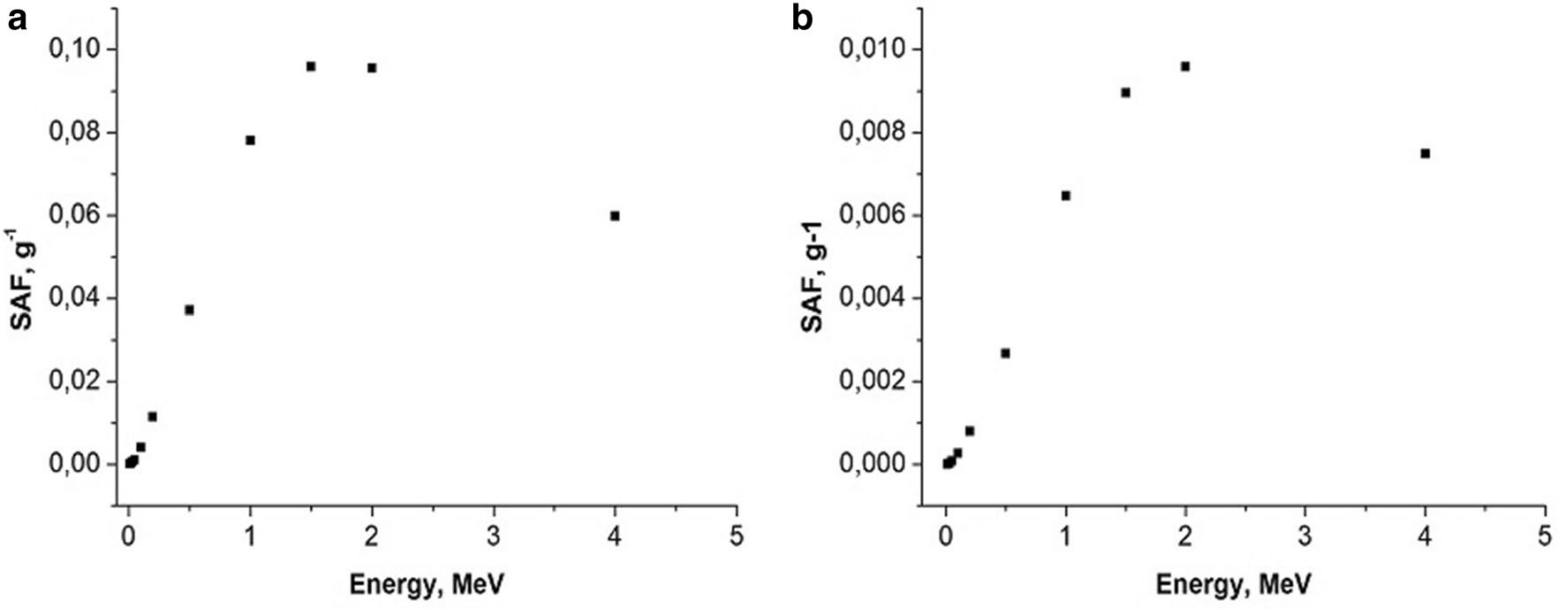
Small intestine irradiating large intestine with electrons as a function of energy, MeV. Left panel: mouse; right panel: rat. Whole body weight of a mouse (**a**): 30 g; whole body weight of a rat (**b**): 270 g; SAF, g^−1^: specific absorbed fraction of electron energy

**Fig. 9:**
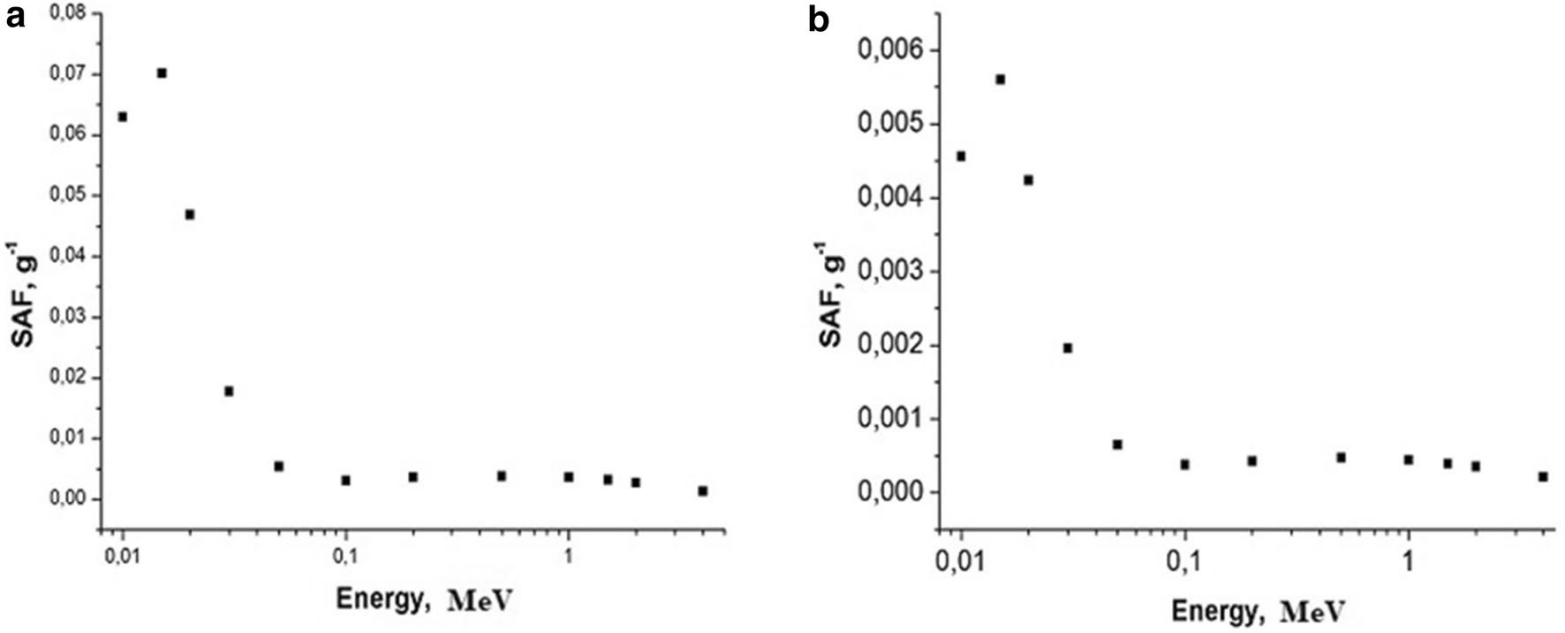
Small intestine irradiating large intestine with gammas as function of energy, MeV. Left panel: mouse, right panel: rat. Whole body weight of a mouse (**a**): 30 g; whole body weight of a rat (**b**): 270 g; SAF, g^−1^: specific absorbed fraction of gamma energy

Examples of calculated specific absorbed fractions (SAF—absorbed fraction of emitted energy per unit of organ’s mass) for gammas and electrons as a function of energy are shown in Figs. 6, 7, 8, 9.

Accumulated doses of internal irradiation were estimated from the beginning of exposure until infinity. It was assumed that physical decay of ^56^Mn was essentially faster than biological redistribution of MnO_2_ powder in the experimental animals. Results of internal dose estimations are presented in Tables 2 and 3.

**Table 2:** Doses of internal irradiation and corresponding standard deviations (*D* ± SD, Gy) in organs of experimental rats resulted from exposure to various activity levels of neutron-activated ^56^MnO_2_ powder

Organs of Wistar rats	Initial activity of 100 mg dispersed ^56^MnO_2_: 2.74 × 10^8^ Bq	Initial activity of 100 mg dispersed ^56^MnO_2_: 5.5 × 10^8^ Bq	Initial activity of 100 mg dispersed ^56^MnO_2_: 8.0 × 10^8^ Bq
	*D* ± SD, Gy	*D* ± SD, Gy	*D* ± SD, Gy
Lungs	0.025 ± 0.004	0.048 ± 0.011	0.065 ± 0.013
Heart	0.0011 ± 0.0002	0.0039 ± 0.0012	0.0083 ± 0.0012
Small intestine	0.17 ± 0.02	0.42 ± 0.07	0.61 ± 0.14
Large intestine	0.29 ± 0.06	0.52 ± 0.11	0.76 ± 0.17
Stomach	0.09 ± 0.01	0.21 ± 0.02	0.30 ± 0.05
Esophagus	0.0069 ± 0.0012	0.016 ± 0.002	0.025 ± 0.006
Liver	0.0015 ± 0.0003	0.0045 ± 0.0012	0.0071 ± 0.0016
Spleen	0.00028 ± 0.00007	0.00050 ± 0.00011	0.00083 ± 0.00019
Kidney	0.00027 ± 0.00006	0.00064 ± 0.00012	0.00098 ± 0.00018
Trachea	0.0058 ± 0.0011	0.0120 ± 0.0024	0.019 ± 0.004
Skin	0.071 ± 0.021	0.110 ± 0.023	0.142 ± 0.028
Eyes	0.019 ± 0.004	0.041 ± 0.008	0.062 ± 0.012

Number**s** of rats in each cage for each irradiation were different, that is, from 6 to 9 (rats) per cage

**Table 3:** Doses of internal irradiation and corresponding standard deviations (*D* ± SD, Gy) in organs of experimental mice resulted from exposure to various activity levels of neutron-activated ^56^MnO_2_ powder

Organs of mice	Initial activity of 100 mg dispersed ^56^MnO_2_: 8 × 10^7^ Bq	Initial activity of 100 mg dispersed ^56^MnO_2_: 2.74 × 10^8^ Bq	Initial activity of 100 mg dispersed ^56^MnO_2_ : 2.74 × 10^8 ^ Bq	Initial activity of 100 mg dispersed ^56^MnO_2_: 2.74 × 10^8^ Bq	Initial activity of 100 mg dispersed ^56^MnO_2_ : 2.74 × 10^8^ Bq	Initial activity of 100 mg dispersed ^6^MnO_2_: 8 × 10^8^ Bq	Initial activity of 100 mg dispersed ^56^MnO_2_: 8 × 10^8^ Bq	Initial activity of 100 mg dispersed ^56^MnO_2_: 8 × 10^8^ Bq
	*D* ± SD, Gy, (C57Bl mice)	*D* ± SD, Gy, (C57Bl mice)	*D* ± SD, Gy, (C57Bl mice)	*D* ± SD, Gy, (BALB/C mice)	*D* ± SD, Gy, (CD-1 mice)	*D* ± SD, Gy, (C57Bl mice)	*D* ± SD, Gy, (C57Bl mice)	*D* ± SD, Gy, (BALB/C mice)
Lungs	0.026 ± 0.005	0.096 ± 0.013	0.14 ± 0.02	0.11 ± 0.03	0.12 ± 0.02	0.25 ± 0.05	0.34 ± 0.07	0.38 ± 0.07
Heart	0.021 ± 0.005	0.056 ± 0.011	0.07 ± 0.01	0.061 ± 0.014	0.089 ± 0.017	0.12 ± 0.02	0.18 ± 0.04	0.15 ± 0.04
Small intestine	0.25 ± 0.09	0.91 ± 0.15	1.1 ± 0.2	0.86 ± 0.21	1.4 ± 0.3	2.3 ± 0.2	2.8 ± 0.4	2.4 ± 0.4
Large intestine	1.2 ± 0.16	4.2 ± 0.5	4.5 ± 0.5	3.8 ± 0.6	3.4 ± 0.5	10.1± 1.4	11 ± 2.1	9.5 ± 2.1
Stomach	0.27 ± 0.08	0.98 ± 0.16	1.2 ± 0.2	0.91 ± 0.22	0.81 ± 0.12	2.4 ± 0.5	2.2 ± 0.3	3.2 ± 0.5
Esopha-Gus	0.032 ± 0.005	0.087 ± 0.013	0.079 ± 0.013	0.093 ± 0.016	0.052 ± 0.011	0.29 ± 0.05	0.17 ± 0.024	0.21 ± 0.04
Liver	0.0018 ± 0.0007	0.0066 ± 0.0011	0.0086 ± 0.0014	0.0076 ± 0.0012	0.0081 ± 0.0016	0.023 ± 0.002	0.022 ± 0.004	0.024 ± 0.005
Spleen	0.0006 ± 0.0001	0.0025 ± 0.0007	0.0028 ± 0.0006	0.0032 ± 0.0008	0.0036 ± 0.0007	0.006 ± 0.001	0.008 ± 0.002	0.007 ± 0.002
Kidney	0.0007 ± 0.0001	0.0028 ± 0.0005	0.0021 ± 0.0006	0.0026 ± 0.0004	0.0023 ± 0.0006	0.007 ± 0.002	0.006 ± 0.002	0.007 ± 0.002
Trachea	0.015 ± 0.004	0.039 ± 0.003	0.047 ± 0.008	0.05 ± 0.01	0.041 ± 0.009	0.14 ± 0.06	0.16 ± 0.04	0.13 ± 0.03
Skin	0.12 ± 0.03	0.29 ± 0.05	0.34 ± 0.06	0.31 ± 0.07	0.42 ± 0.09	0.96 ± 0.21	0.91 ± 0.16	0.99 ± 0.23
Eyes	0.041 ± 0.009	0.14 ± 0.05	0.13 ± 0.02	0.16 ± 0.03	0.12 ± 0.03	0.39 ± 0.08	0.32 ± 0.07	0.34 ± 0.07

Numbers of mice in each cage for each irradiation were different, that is, from 3 to 10 animals per cage

## Discussion

In the present study, we found that under similar exposure conditions to ^56^MnO_2_ powder, the internal doses in mice were several times higher in comparison with rats. This can, perhaps, be explained by the following: higher breathing rate in mice versus rats and, lower organ weight in mice compared with rats (Besyadovsky et al. 1978). It should be noted that the latter circumstance leads to the fact that the specific absorbed fraction of energy (that is, fraction of absorbed energy per unit mass of the organ) is essentially higher in mice than in rats (see Figs. 6, 7, 8, 9). Difference in doses of internal irradiation of mice with the same activity of ^56^MnO_2_ powder dispersed over the experimental animals can be explained by the fact that the number of mice per cage was different during different irradiation sessions (see Table 3 with corresponding note). This can also explain the absence of a simple proportionality between the internal radiation doses and the dispersed activity of ^56^MnO_2_ (Tables 2 and 3). The increased doses in the lungs are explained by the fact that this organ is critical when inhaling small radioactive particles of ^56^MnO_2_, which leads to an increased accumulation of activity in this organ. High doses of irradiation of the gastrointestinal tract can be explained by the fact that in the process of cleaning and grooming, experimental animals swallowed radioactive particles retained by their hair, which led to a high accumulation of activity in the stomach and intestines during exposure (1 h), as it was noted in Stepanenko et al. (2017), Shichijo et al. (2017). The retention of radioactive particles by animal hair leads to an increase in skin radiation dose.

## Conclusion

This study aimed to estimate internal doses in laboratory animals (mice and rats) that had been exposed to various levels of ^56^MnO_2_ in the form of dispersed powder. The experiment was performed in support of the Japanese initiative to investigate the biological effects of irradiation from residual neutron-activated radioactivity that resulted from the A-bombing (Hoshi 2020; Roesch 1987; Imanaka et al. 2012; Kerr et al. 2013, 2015; Ohtaki et al. 2014). Radionuclide ^56^Mn (T_1/2_ = 2.58 h) is one of the main neutron-activated emitters during the first hours after neutron activation of soil dust particles.

In our previous studies (Stepanenko et al. 2017; Shichijo et al. 2017) related to irradiation of male Wistar rats after dispersion of ^56^MnO_2_ powder, the internal doses in rats were found to be very inhomogeneous: distribution of doses among different organs ranged from 1.3 Gy in small intestine to less than 0.0015 Gy in some of the other organs. Internal doses in the lungs ranged from 0.03 to 0.1 Gy. The essential pathological changes were found in lung tissue of rats despite a low level of irradiation.

In the present study, the dosimetry investigations were extended: internal doses in experimental mice and rats were estimated for various activity levels of dispersed neutron-activated ^56^MnO_2_ powder.

The following findings were noted:

- Internal radiation doses in mice were several times higher in comparison with rats under similar conditions of exposure to ^56^MnO_2_ powder.
- When 2.74 × 10^8^ Bq of ^56^MnO_2_ powder was dispersed over mice, doses of internal irradiation ranged from 0.81 to 4.5 Gy in the gastrointestinal tract (small intestine, stomach, large intestine), from 0.096 to 0.14 Gy in lungs, and doses in skin and eyes ranged from 0.29 to 0.42 Gy and from 0.12 to 0.16 Gy, respectively. Internal radiation doses in other organs of mice were much lower.
- Internal radiation doses were significantly lower in organs of rats with the same activity of exposure to ^56^MnO_2_ powder (2.74 × 10^8^ Bq): 0.09, 0.17, 0.29, and 0.025 Gy in stomach, small intestine, large intestine, and lungs, respectively.
- Doses of internal irradiation in organs of rats and mice were two to four times higher when they were exposed to 8.0 × 10^8^ Bq of ^56^MnO_2_ (in comparison with exposure to 2.74 × 10^8^ Bq of ^56^MnO_2_).
- Internal radiation doses in organs of mice were 7–14 times lower with the lowest ^56^MnO_2_ amount (8.0 × 10^7^ Bq) in comparison with the highest amount, 8.0 × 10^8^ Bq, of dispersed ^56^MnO_2_ powder.

The data obtained will be used for interpretation of biological effects in experimental mice and rats that result from dispersion of various levels of neutron-activated ^56^MnO_2_ powder, which is the subject of separate studies.
